# The clinical significance of radiological changes associated with gliadel implantation in patients with recurrent high grade glioma

**DOI:** 10.1038/s41598-022-27128-4

**Published:** 2023-01-02

**Authors:** Oz Haim, Ariel Agur, Or-Tal Efrat, Pablo Valdes, Zvi Ram, Rachel Grossman

**Affiliations:** Department of Neurosurgery, Tel-Aviv Medical Center, affiliated to the Sackler Faculty of Medicine, Tel-Aviv University, 6 Weizman Street, 6423906 Tel-Aviv, Israel

**Keywords:** CNS cancer, Brain, Surgical oncology, Cancer imaging

## Abstract

Gliadel occasionally induces edema following its implantation. We aimed to correlate such post-surgical radiological changes to its efficacy and subsequent survival. Fifty-six patients with recurrent high grade glioma were treated between 2005 and 2016 with Gliadel implantation. Volumetric measurements of MRI features, including FLAIR abnormalities, tumor bulk (volume of gadolinium enhancement on T1) and resection cavity volumes over time were conducted. To assess dynamics over time, linear regression trendlines for each of these were calculated and examined to correlate with survival. Median follow-up after resection was 21.5 months. Median survival post-Gliadel implantation and overall survival since diagnosis were 12 months and 22 months, respectively. A subgroup of patients (n = 6) with a transient increase in FLAIR changes volume over time survived significantly longer post-Gliadel compared to those who did not demonstrate such change (36 vs 12 months, *p* = .03). Positive trends, representing overall growth in volume over time, of tumor bulk and resection cavity predicted survival in multivariate analyses (hazard ratios 7.9 and 84, *p* = .003 and .002, respectively). Increase in tumor bulk and resection cavity over time were associated with decreased survival, while transient FLAIR increase was a favorable prognostic factor. This may represent a transient inflammatory process in the tumor, possibly stemming from a presumed immune-mediated anti-tumor response.

## Importance of the study

Treatment of recurrent high-grade gliomas is limited, with Gliadel wafer implantation being one with proven efficacy in certain patient populations. In this study we describe the dynamics of MRI features associated with Gliadel implantation, assessed volumetrically, and analyzed these over time. These features were found to correlate with survival. We have also assessed a transient increase in FLAIR changes (representative of inflammatory parenchymal reaction to the Gliadel wafers) allows to stratify patients early on to assess for better response to Gliadel, representing an imaging-based prognosticator.

## Introduction

There is currently no standard therapeutic protocol for patients with recurrent high-grade glioma (HGG) after failure of initial treatment, and the therapeutic options are limited to re-operation in selected cases, a second line of chemotherapy, re-irradiation, and biological treatments. Carmustine wafers (Gliadel) are a biodegradable polymer containing high concentrations of BCNU (7.7 mg each) and constitute the most studied type of implantable chemotherapy wafer for HGG^1–4^. Up to 8 wafers are placed in the resection cavity after tumor resection. Pharmacological studies have shown that the Gliadel wafers release BCNU in vivo over a period of 21 days, mostly during the first 5–7 days, and are completely degraded at 6–8 weeks after implantation following renal elimination^5,6^. Theoretically, local delivery of chemotherapy may be superior to systemic administration, taking into consideration the blood–brain-barrier’s low permeability, higher local concentration of the drug at the tumor site, reduced systemic toxicity,^7^ and local cell death that may, in turn, stimulate an anti-tumor inflammatory response^8,9^. The role of Gliadel wafers in the treatment of HGG, whether primary or recurrent, was established by many studies that suggested improvement in overall survival^1,3,10,11^, especially within specific subgroups, such as O(6)-methylguanine-methyltransferase (MGMT) methylated tumors^12^. Along with the advantages, however, the use of Gliadel harbors some adverse effects, including peritumoral edema, infection, cyst formation in the resection cavity, wound healing complications, CSF leak, and postoperative meningitis^13,14^. These sequelae account for less than 1% of all major complications and rarely necessitate removal of the Gliadel wafers^13^.

The presence of Gliadel wafers may change various characteristics of postoperative magnetic resonance imaging (MRI) studies with regard to time span^15–17^. MRI characteristics include tumor bulk (TB), surrounding edema (as evident by fluid-attenuated inversion recovery [FLAIR] abnormalities [FA]) and resection cavity (RC). Progression of surrounding edema has already been observed by Aoki et al. in up to 25% of patients^18^. Theoretically, these changes may be the result of Gliadel-induced anti-tumor inflammatory activity^16,19^ that can potentially affect survival via immune-mediated processes^20,21^.

In this study, we assessed the association between MRI characteristics (TB, FA, and RC) and clinical outcomes in patients with recurrent HGG who underwent re-operation for tumor resection and implantation of Gliadel wafers. We hypothesized that changes related to the Gliadel wafers in the follow-up MRI scans may be an indicator of anti-tumor inflammatory response, and thus might correlate with clinical outcomes.

## Methods

### Data collection

Potential study patients were identified from the archives of the Department of Neurosurgery of the Tel-Aviv Medical Center (TLVMC) between January 2005 and December 2016. Included were all the patients above 18 years of age who were re-operated for the resection of recurrent HGG and implantation of Gliadel wafers. Patients who lacked sufficient clinical preoperative and postoperative data or MRI scans (at least 2 postoperative and 1 preoperative scans), or whose MRI scans were carried out too long after the surgery date (more than 8 months) were excluded, as well as patients with any sign for radiation necrosis or inflammation in the pathological reports. A total of 56 suitable patients were included in the final analysis. Of note, immediate postoperative MRI imaging was available for 33 (of the 56) patients who underwent surgery after the imaging protocol had been modified in our department in 2011, mandating postoperative imaging in all patients within 48 h after surgery.

The extracted parameters included age, sex, hand dominance, presenting symptoms, and Karnofsky Performance Scale (KPS) score at the time of recurrence, location of the recurrent tumor, use of steroids preoperatively, postoperative complications (e.g., wound infection, hemorrhage, or neurologic deterioration), readmissions during the first three month postoperatively (and their causes), and dates of deaths. All pathologic reports were performed by the Department of Pathology of TLVMC, based upon World Health Organization criteria^22^. The data on isocitrate dehydrogenase (IDH) and MGMT statuses were retrieved when available. Data on 1p/19q co-deletion was unavailable to any of our patients.

**Figure 1:**
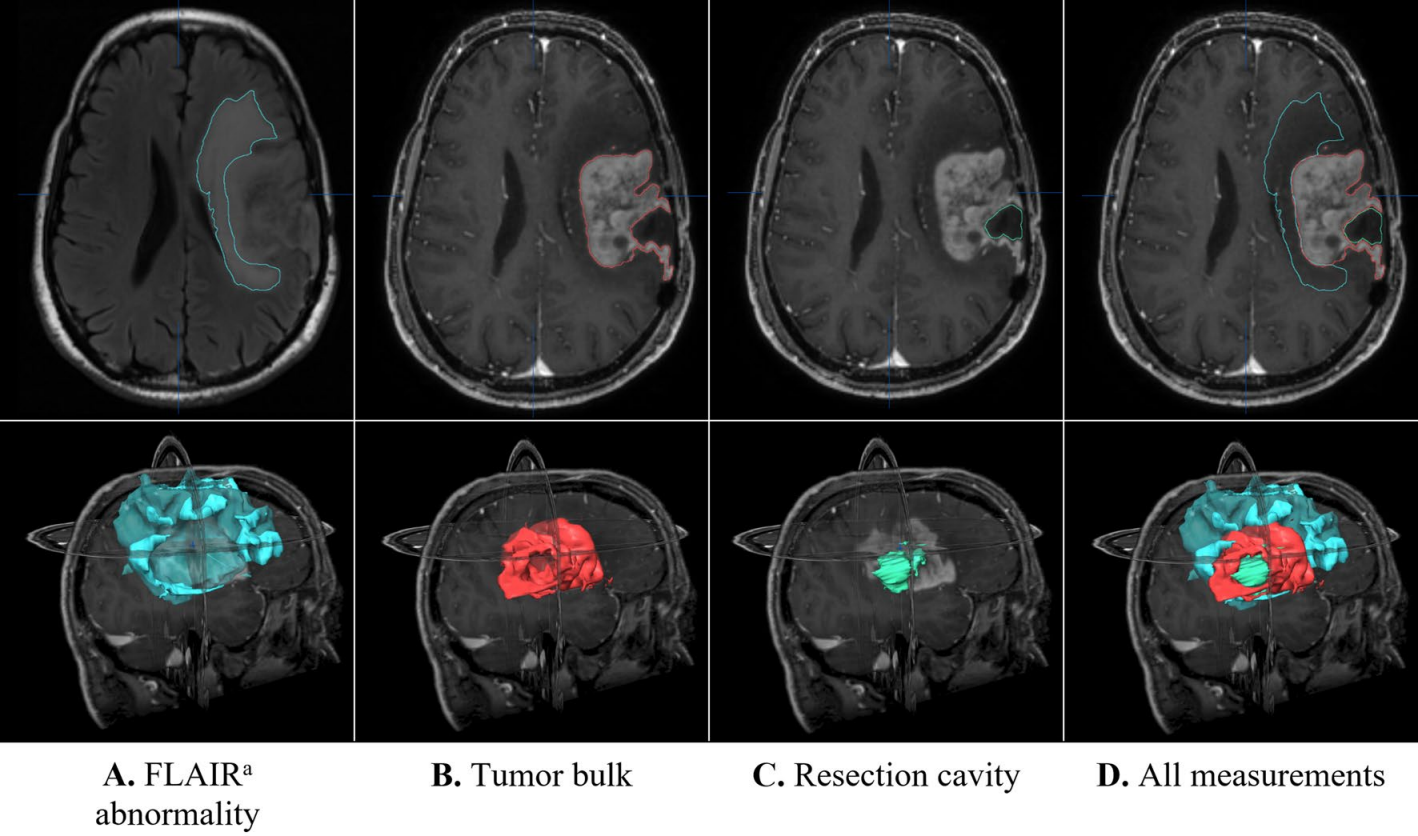
Volumetric measurements of postoperative MRI imaging. All volumetric measurements were done by using BrainLab IPlannet 3.0 software (Brainlab AG, Munich, Germany). Pre-operative measurements of FLAIR^a^ T2-weighted (FLAIR^a^ abnormality) and contrast-enhanced T1-weighted magnetic resonance imaging (tumor bulk and resection cavity) in a patient with recurrent HGG with prior tumor resection who underwent a repeat resection with Gliadel wafers. ^a^T2-Weighted fluid-attenuated inversion recovery.

We measured 3 volumetric parameters on each MRI scan, TB (tumor bulk), FLAIR T2 abnormality (FA), and resection cavity (RC: available only in postoperative studies). The usefulness of semi-automatic measurements of tumor volumes is well established^23,24^, especially when done by a single observer^25^. Accordingly, our measurements were carried out semi-automatically on axial sections by means of BrainLab IPlannet 3.0 software (Brainlab AG, Munich, Germany) (Fig. 1), and all measurements were carried out by a single neurosurgeon (O.H.), thereby avoiding inter-observer differences. The preoperative tumor volume and peritumoral edema were measured with T1-weighted gadolinium-enhanced and FLAIR T2 sequences, respectively.

The study protocol was approved by the institutional review board (IRB) in Tel-Aviv Medical Center (IRB approval number 0478-17-TLV). Due to its retrospective nature, no informed consent was required by the IRB for this study. The study protocol was carried out in accordance with all the relevant guidelines and regulations.

### Statistical analysis

The data were analyzed by the R statistical programming language. Significance was set at a *p*-value of < 0.05. Descriptive analysis is reported with mean, median and standard error (SE) for continuous variables, and with frequency distribution for categorical variables. Overall survival (OS) was defined and calculated as the time from the date of first surgery to the date of death. Gliadel survival (GS) was defined and calculated in the same manner using the date of Gliadel implantation as the first date. The date of last follow-up was reported in the absence of the date of death. A linear regression trendline was calculated for every volumetric parameter with the use of the sum of the least squares technique. The slope of the trendline was then used as a continuous variable for each patient and each parameter, representing the change of volume over time.

Survival analyses were conducted to delineate parameters associated with OS and GS. Kaplan–Meier plots were used to estimate the event-free survival using the Mantel Cox log-rank test as a statistic. A Cox proportional hazards model was employed for the multivariate analysis with a 95% confidence interval (95% CI) in an all-inclusive and stepwise fashion.

### Previous presentations

Israeli Neurosurgical Society Annual Meeting 2019 Abstract Presentation.

## Results

### Demographics

Fifty-six patients with full data sets were included in the analysis out of 69 records of potentially suitable study enrollees. Their demographic, clinical, and volumetric characteristics are summarized in Table 1. The median follow-up duration was 21.5 months after diagnosis and 12 months after the Gliadel surgery. The mean ± SE age at Gliadel surgery was 51.8 ± 1.8 (range 19–77) years, with a slight male predominance (62.5%). The preoperative KPS was available for 40 patients, 39 of whom had KPS > 70. Twenty patients were treated with steroids before undergoing the Gliadel surgery, consisting of a mean daily dose of 6.5 ± 1.2 mg of dexamethasone. Thirty-six patients (64.3%) had a left-sided tumor. The most common cause for admission for surgery was radiological recurrence without any associated neurologic symptoms (n = 22, 39.3%). Among symptomatic patients at the time of Gliadel surgery, headache and speech deficits (17.8% and 16.1%, respectively) were the most common. Some patients had more than one symptom at presentation.

**Table 1:** Demographics and clinical characteristics of 56 patients who underwent implantation of gliadel wafers for recurrent glioblastoma between 2005 and 2016.

Parameter	Value				
**Age at presentation, years (mean ± SE)**	50.2 ± 1.8				
**Age at Gliadel surgery, years (mean ± SE)**	51.8 ± 1.8				
**Sex, n (%)**					
Male	35 (62.5%)				
Female	21 (37.5%)				
**Karnofsky performance scale (KPS), n (%)**					
≥ 70	39 (69.6%)				
< 70	1 (1.8%)				
Unavailable	16 (28.6%)				
**Receiving steroids before Gliadel surgery, n (%)**	20 (36%)				
**IDH status, n (%)**	42 (75%)				
Positive, n (% of patients with available IDH status)	5 (11.9%)				
Negative, n (% of patients with available IDH status)	37 (88.1%)				
**Presentation, n (%)**					
Seizure	6 (10.7%)				
Headache	10 (17.8%)				
Visual deficit	5 (8.9%)				
New motor complaints	4 (7.1%)				
New speech complaints	9 (16.1%)				
Others (cognitive, fatigue, nausea and vomiting, etc.)	16 (28.6%)				
No symptoms (radiological progression)	22 (39.3%)				
**Location, n (%)**^**a**^	Left (36, 64.3%)			Right (20, 36%)	
Frontal	13 (23.2%)			7 (12.5%)	
Parietal	8 (14.3%)			9 (16.1%)	
Temporal	11 (19.6%)			5 (8.9%)	
Occipital	5 (8.9%)			2 (3.6%)	
**Surgeries prior to resection with Gliadel, n (%)**					
One	52 (92.9%)				
Two	4 (7.1%)				
**Interval between 1st resection to Gliadel, months (mean ± SE)**	14 ± 2.1				
**Volume of preoperative FLAIR abnormality, cm**^**3**^ **(mean ± SE)**	54.8 ± 5.3				
**Volume of preoperative tumor bulk, cm**^**3**^ **(mean ± SE)**	21.1 ± 2.8				
**Interval between resection to immediate postoperative imaging test, days (mean ± SE)**	1.2 ± 0.1				
**Extent of resection**^**b**^ **(mean ± SE)**	93.9 ± 0.9				
**Pathology of first resection, n (%)**	WHO 2	WHO 3			WHO 4
Oligodendroglioma	1 (1.8%)				
Anaplastic astrocytoma		6 (10.7%)			
Anaplastic oligodendroglioma		2 (3.6%)			
Anaplastic pleomorphic xanthoastrocytoma		1 (1.8%)			
Glioblastoma, NOS					46 (82.1%)
Pathology of resection with Gliadel, n (%)	WHO 2		WHO 3		WHO 4
Anaplastic astrocytoma			1 (1.8%)		
Anaplastic oligodendroglioma			2 (3.6%)		
Anaplastic pleomorphic xanthoastrocytoma			1 (1.8%)		
Glioblastoma, IDH-wildtype					32 (57.1%)
Astrocytoma, IDH-mutant					2 (3.6%)
Glioblastoma, NOS					12 (21.4%)
**Postoperative complications, n (%)**	20 (35.7%)				
Deep infection^c^	10 (17.9%)				
Superficial wound dehiscence^d^	2 (3.6%)				
Hemorrhage	3 (5.4%)				
Neurological deterioration	10 (17.9%)				
Other (chemical meningitis, decreased consciousness, respiratory, etc.)	3 (5.4%)				
**Readmission within 1 month, n (%)**	24 (42.9%)				
Infection^e^	7 (12.5%)				
Wound^f^	9 (16.1%)				
Neurological	5 (8.9%)				
Other	12 (21.4%)				
Readmission within 2–3 months	15 (26.8%)				
Readmission within 2–3 months, n (%)	23 (41.1%)				
Readmission within 1 month	15 (26.8%)				
Infection^g^	4 (7.1%)				
Wound^h^	7 (12.5%)				
Neurologic	8 (14.3%)				
Other	14 (25%)				
**Survival**					
Alive at date of statistical analysis, n (%)	6 (10.7%)				
Survival since Gliadel surgery, months (median ± SE)	12 ± 3.8				
Overall survival, months (median ± SE)	22 ± 4				

*SE* standard error.

^a^Some patients had more than 1 lobe involvement.

^b^Given in percent, calculated on TB volumes between pre-operation and immediately post-operation (35 patients).

^c^Meningitis, osteomyelitis, abscess (including readmission in 1 month).

^d^Wound dehiscence, CSF leak, pseudo-meningocele requiring hospitalization (during immediate postoperative period).

^e,f^During readmission within 1 month post-operation.

^g^1 of them was infected during the immediate postoperative period.

^h^3 of them had wound pathologies during the immediate postoperative period.

The mean time between the first surgery and the Gliadel surgery was 14 ± 2.1 months. Four patients (7.1%) underwent Gliadel implantation during the third resection (i.e., the second recurrence). The mean extent of tumor resection (EOR) was 93.9% ± 0.9%, calculated among the 33 patients who had available immediate postoperative imaging. Forty-two patients had IDH mutation status, of whom five had a mutation. MGMT methylation status was available for only three patients and that parameter was therefore not included in the final analysis. Twenty patients (35.7%) had at least one postoperative complication. The most common complications were neurological deterioration and deep surgical infections (17.9% each).

Adjuvant salvage therapies were given to patients at the discretion of a neuro-oncologist, with 39 patients (69.6%) receiving them. They were comprised of bevacizumab (n = 36, 64.3%), radiotherapy (n = 14, 25%), temozolomide (n = 10, 17.9%), and other chemotherapeutical agents (n = 10, 17.9%). Some patients received more than one adjuvant treatment. The use of adjuvant therapies did not correlate significantly with survival (*p* = 0.54).

The mean interval between postoperative imaging tests was 58 ± 2 days. Fifty patients (89.3%) had three imaging tests postoperatively, six had two imaging tests postoperatively. All imaging tests were analyzed volumetrically as described above. The median OS of the entire cohort was 22 ± 4 months. The median survival after the resection with Gliadel (GS) was 12 ± 3.8 months.

### Clinical characteristics and survival

In the univariate analysis (Table 2), age was the only clinical parameter significantly associated with survival, with a median survival from implantation of Gliadel (GS) of 10 months in patients ≥ 60 years of age compared to 13 months in patients < 60 years of age (*p* = 0.04). There was a trend toward significance for IDH status and KPS ≥ 70 at last follow-up (*p* = 0.13 and 0.06, respectively).

**Table 2:** Univariate and multivariate analyses of clinical and volumetric characteristics and survival of 56 patients who underwent implantation of Gliadel wafers for recurrent glioblastoma between the years 2005–2016.

	Univariate				All-inclusive multivariate (n = 53)			Stepwise multivariate (n = 53)		
Parameter	MS	HR	95% CI	*p*-value	HR	95% CI	*p*-value	HR	95% CI	*p*-value
**Age, y (n = 56)**								Left out of model		
< 60	13									
≥ 60	10	1.9	1.02–3.4	.04 *	1.02	0.99–1.05	.27			
**Sex (n = 56)**								Left out of model		
Male	12									
Female	11	1.2	0.7–2	.61	1.6	0.7–3.3	.25			
**Number of recurrences (n = 56)**								Left out of model		
1	12									
2–3	9.5	0.9	0.4–2	.86	2.1	0.8–5.3	.11			
**Symptoms at presentation (n = 56)**								Left out of model		
Yes	12	0.7	0.4–1.3	.31	1.2	0.5–2.9	.69			
No	12.5									
**Pre-op FLAIR abnormality (n = 56)**		1	0.99–1.01	.48	1	0.99–1.01	.39	Left out of model		
≥ 43.3 cm^3^ (median)	12									
< 43.3 cm^3^ (median)	12									
**Pre-op tumor bulk (n = 56)**		1.01	0.99–1.03	.06	1.01	0.99–1.03	.32	Left out of model		
≥ 15.3 cm^3^ (median)	11									
< 15.3 cm^3^ (median)	13.5									
**Extent of resection (n = 33)**		0.99	0.95–1.05	.83	Not included in model			Not included in model		
≥ 95%	12									
< 95%	6									
**Any complications (n = 54)**								Left out of model		
Yes	11.5	0.98	0.6–1.8	.96	2.1	0.7–6.3	.2			
No	12									
**Infectious complications (n = 56)**					0.6	0.2–2	.37	Left out of model		
Yes	9	1.3	0.7–2.6	.51						
No	12									
**Neurologic complications (n = 53)**								Left out of model		
Yes	20	0.6	0.3–1.3	.17	0.2	0.06–0.8	.026 *+			
No	11									
**Readmission within 1 month (n = 56)**								Left out of model		
Yes	9.5	1.4	0.8–2.5	.23	1.3	0.6–3	.48			
No	13									
**Readmission 2–3 months (n = 56)**								Left out of model		
Yes	10	1.6	0.9–2.8	.1	1.2	0.5–3	.7			
No	13									
**WHO grade (Gliadel surgery) (n = 56)**								Left out of model		
WHO 3	10									
WHO 4	12	1.1	0.3–3.5	.92	0.6	0.1–3.2	.53			
**IDH status (n = 41)**					Not included in model			Not included in model		
IDH mutant	20	0.4	0.2–1.3	.13						
IDH wildtype	12									
**KPS at last follow-up (n = 36)**					Not included in model			Not included in model		
≥ 70	20	0.4	0.2–1.02	.06						
< 70	12									
**Post-op FLAIR abnormality (n = 56)**					1.01	0.5–2.3	.97	Left out of model		
Increased (slope > 0)	10	1.9	1.1–3.3	.03 *						
Decreased (slope ≤ 0)	14									
**Post-op tumor bulk (n = 56)**					7.9	2–31.5	.003 **	7.3	3.4–15.7	3∙10^–7^ ***
Increased (slope > 0)	11	3.1	1.5–6.5	.003 **						
Decreased (slope ≤ 0)	27									
**Post-op surgical cavity (n = 56)**					84	5.3–1318.5	.002 **	Left out of model		
Increased (slope > 0)	6	3.8	1.9–7.5	.0002 ***						
Decreased (slope ≤ 0)	13									

MS, median survival in months; HR, Hazard ratio; CI, confidence interval; FLAIR, T2 Weighted fluid-attenuated inversion recovery; WHO, World health organization; IDH, Isocitrate dehydrogenase; KPS, Karnofsky performance scale.

*p < .05, **p < .01, ***p < .001.

+Wald’s *p*-value.

### Dynamics of volumetric measurements and survival

**Figure 2:**
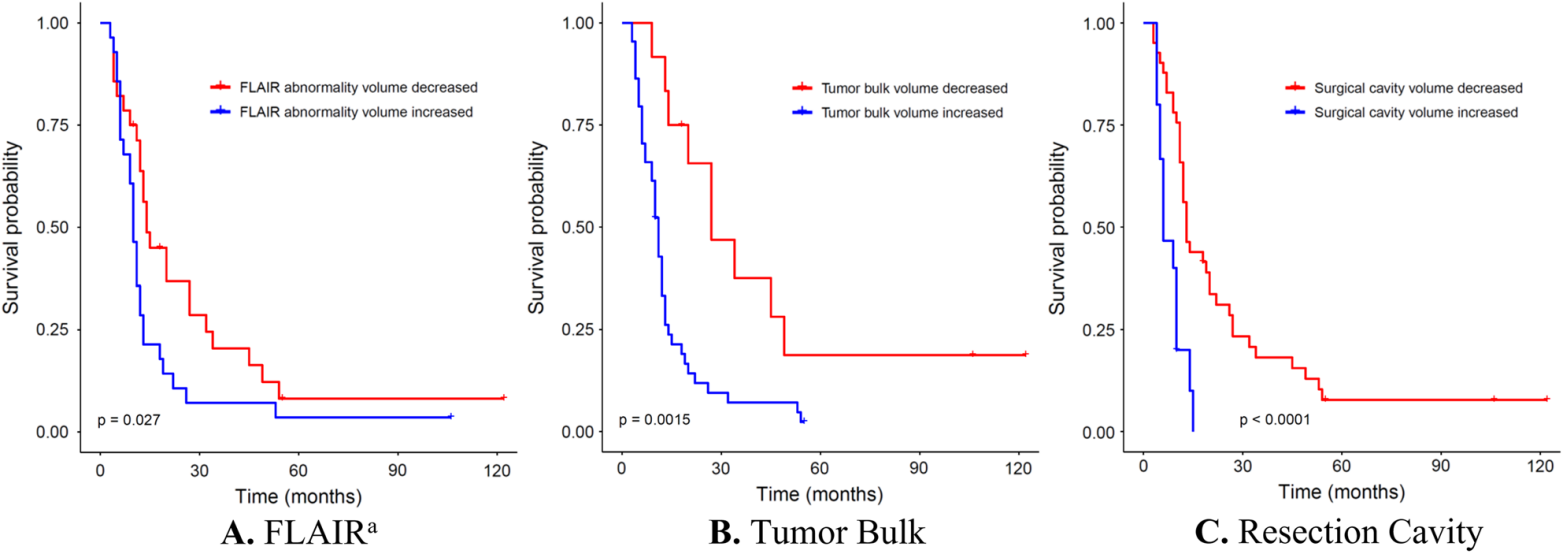
Kaplan–Meier survival curves for volumetric measurements in patients who underwent resection of tumor recurrence with implantation of Gliadel wafers. ^a^T2-Weighted fluid-attenuated inversion recovery.

Increasing volumes of postoperative FA, TB, and RC all correlated negatively with Gliadel survival in a univariate analysis (Table 2). Patients with increasing volume of FA over time had a median GS of 10 months compared to 14 months for patients whose FA volume was decreasing (hazard ratio [HR] = 1.9, *p* = 0.03). Likewise, patients with increasing TB volume over time had a median GS of 11 months compared to 27 months for patients with decreasing TB volume (HR = 3.1, *p* = 0.003). Similarly, RC volume trends correlated with survival. GS of 6 months was seen in patients whose RC volume increased, compared to 13 months for those whose RC volume decreased (HR = 3.8, *p* = 0.0002). Kaplan–Meier survival plots of these analyses are presented in Fig. 2.

In an all-inclusive multivariate analysis (Table 2), FA no longer predicted survival. However, both an increased TB and RC significantly correlated with worse survival (HR = 7.9, *p* = 0.003; HR = 84, *p* = 0.002, respectively). Goodness-of-fit for the model was estimated at a concordance of 79%. In a stepwise multivariate analysis, the only parameter associated with survival was the volume of TB, with an HR of 7.3 and Wald’s *p*-value set at 3∙10^–7^ (95% confidence interval 3.4–15.7). Goodness-of-fit for the stepwise model was estimated at a concordance of 73% (Table 2).

### Temporary FLAIR reactivity effect on survival

Gliadel may induce a considerable amount of peritumoral edema^16,19^, which may represent anti-tumor inflammatory activity in a selected group of patients^20,21^. Accordingly, we analyzed a subgroup of patients who had a temporary surge of peritumoral edema, presenting as increase in FA with subsequent decrease of FA in follow-up imaging. The beginning of this period was set at postoperative day 21 in order to allow for postoperative changes and for direct toxicity of BCNU to resolve^5^. Accordingly, the end of this period was set to 8 weeks to fit the known stabilization of MRI findings after Gliadel implantation, at two months postoperatively^17^. In order to define a significant and reliable degree of change of the FLAIR abnormalities, we set a threshold of 20%. This is double the published intra-observer reliability for contrast enhanced T1 images^25^, and stems from the fact that FLAIR changes are less well defined than enhanced T1 images. Patients who had > 20% increase of FA volume between 3–8 weeks post-surgery with subsequent resolution or > 20% decrease in FA were considered as having a “Reactive FLAIR”.

**Figure 3:**
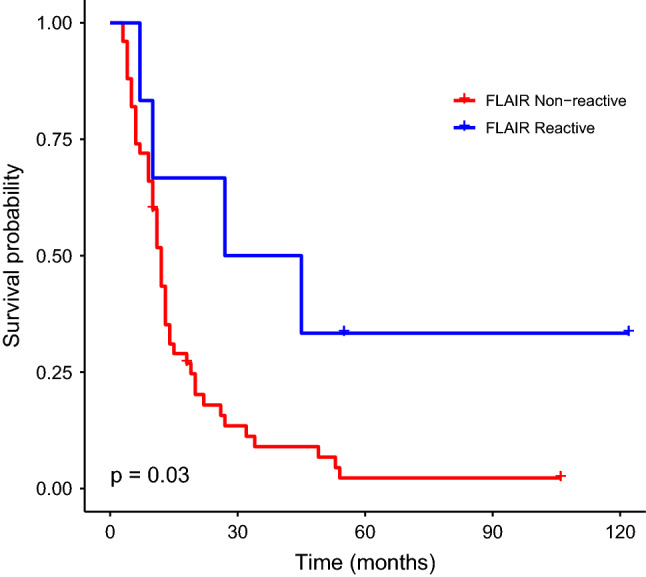
Kaplan–Meier survival plot comparing survival between group with FLAIR^a^ reactivity vs. non-reactivity. ^a^ T2-Weighted fluid-attenuated inversion recovery.

Six of the 56 patients (10.7%) belonged to this group, while the rest of the cohort served as control (thus defined “Non-reactive FLAIR”). Table 3 summarizes the basic characteristics of these two groups. No significant differences were found in preoperative tumor and patient characteristics and postoperative adjuvant treatments. The median survival after Gliadel surgery for patients who had a reactive FLAIR was 36 months compared to 12 months for the non-reactive FLAIR group (*p* = 0.003) (Fig. 3).

**Table 3:** Demographic and survival characteristics of 56 patients who underwent implantation of Gliadel Wafers for Recurrent Glioblastoma between 2005 and 2016, stratified by FLAIR reactivity.

Parameter	FLAIR non-reactive n = 50	FLAIR reactive n = 6	*p*-value
**Age at presentation, years (mean ± SE)**	50.2 ± 1.9	49.5 ± 5	.9
**Age at Gliadel surgery, years (mean ± SE)**	51.9 ± 1.9	51.5 ± 4.6	.95
**Sex, n (%)**			.12
Male	29 (58%)	6 (100%)	
Female	21 (42%)	0 (0%)	
**Karnofsky Performance Scale (KPS), n (%)**			1
≥ 70	35 (70%)	4 (67%)	
< 70	1 (2%)	0 (0%)	
Unavailable	14 (28%)	2 (33%)	
**Receiving steroids before Gliadel surgery, n (%)**			.74
Yes	16 (32%)	2 (33.3%)	
No	25 (50%)	1 (16.7%)	
Unavailable	9 (18%)	3 (50%)	
**IDH status, n (%)**			.98
Positive	4 (8%)	1 (16.7%)	
Negative	33 (66%)	3 (50%)	
Unavailable	13 (26%)	2 (33.3%)	
**Presenting symptoms, n (%)**	29 (58%)	5 (83%)	.45
Seizure	5 (10%)	1 (16.7%)	1
Headache	8 (16%)	2 (33.3%)	.63
Visual deficit	4 (8%)	1 (16.7%)	1
New motor complaints	4 (8%)	0 (0%)	1
New speech complaints	9 (18%)	0 (0%)	.59
None (radiological progression)	21 (42%)	1 (16.7%)	.45
**Location, n (%)**			.98
**Left**			
Frontal	12 (24%)	1 (16.7%)	
Parietal	7 (14%)	1 (16.7%)	
Temporal	9 (18%)	2 (33.3%)	
Occipital	4 (8%)	1 (16.7%)	
**Right**			
Frontal	6 (12%)	1 (16.7%)	
Parietal	9 (18%)	0 (0%)	
Temporal	5 (10%)	0 (0%)	
Occipital	2 (4%)	0 (0%)	
**Number of surgeries prior to resection with Gliadel, n (%)**			1
One	46 (92%)	6 (100%)	
Two	4 (8%)	0 (0%)	
**Interval from 1st resection to Gliadel, months (mean ± SE)**	13.6 ± 2.1	17.6 ± 7.8	.55
**Volume of preoperative FLAIR abnormality, cm**^**3**^ **(mean ± SE)**	58.1 ± 5.7	27.4 ± 5.3	.07
**Volume of preoperative tumor bulk, cm**^**3**^ **(mean ± SE)**	23 ± 3.1	5.3 ± 1.8	.053
**Extent of resection, precents (mean ± SE)**	93.8 ± 1	95.2 ± 0.1	.77
**Pathology of first resection, n (%)**			.83
Oligodendroglioma	1 (2%)	0 (0%)	
Anaplastic astrocytoma	6 (12%)	0 (0%)	
Anaplastic oligodendroglioma	2 (4%)	0 (0%)	
Anaplastic pleomorphic xanthoastrocytoma	1 (2%)	0 (0%)	
Glioblastoma	40 (80%)	6 (100%)	
**Pathology of resection with Gliadel, n (%)**			.92
Anaplastic astrocytoma	1 (2%)	0 (0%)	
Anaplastic oligodendroglioma	2 (4%)	0 (0%)	
Anaplastic pleomorphic xanthoastrocytoma	1 (2%)	0 (0%)	
Glioblastoma	46 (92%)	6 (100%)	
**Postoperative complications, n (%)**	18 (37.5%)	3 (50%)	1
Deep infection	3 (6%)	0 (0%)	
Superficial wound dehiscence	2 (4%)	0 (0%)	
Hemorrhage	3 (6%)	0 (0%)	
Neurological deterioration	8 (16%)	2 (33.3%)	
Other (chemical meningitis, decreased consciousness, respiratory, etc.)	5 (10%)	1 (16.7%)	
**Adjuvant therapies, n (%)**			
Any	36 (72%)	3 (50%)	.52
Avastin	33 (66%)	3 (50%)	.75
Temodal	8 (16%)	2 (33.3%)	.63
Other chemotherapy	8 (16%)	0 (0%)	.66
FSR/SRS	11 (22%)	2 (33.3%)	.91
**Readmission within 1 month, n (%)**	22 (44%)	2 (33.3%)	.95
Infection	7 (14%)	0 (0%)	
Wound	8 (16%)	1 (16.7%)	
Neurological	4 (8%)	1 (16.7%)	
Other	4 (8%)	6 (100%)	
Readmission within 2–3 months	14 (28%)	1 (16.7%)	
**Readmission within 2–3 months, n (%)**	21 (42%)	2 (33.3%)	1
Readmission within 1 month	14 (28%)	1 (16.7%)	
Infection	3 (6%)	1 (16.7%)	
Wound	6 (12%)	1 (16.7%)	
Neurologic	7 (14%)	1 (16.7%)	
Other	5 (10%)	0 (0%)	
**Volumetric trends, n (%)**			
Increased slope of tumor bulk	41 (82%)	3 (50%)	.2
Increased slope of FLAIR abnormalities	26 (52%)	2 (33.3%)	.67
Increased slope of resection cavity	14 (28%)	1 (16.7%)	.92
**Survival, months (median ± SE)**			
Survival since Gliadel surgery	12 ± 2.9	36 ± 19.9	0.03 *
Overall survival	21 ± 3.5	53 ± 18.9	0.042 *

SE, standard error FLAIR, T2 Weighted fluid-attenuated inversion recovery; IDH, Isocitrate dehydrogenase; KPS, Karnofsky performance scale. FSR, Fractionated stereotactic radiotherapy; SRS, Stereotactic radiosurgery

*p < .05, **p < .01, ***p < .001.

## Discussion

The implantation of Gliadel wafers in the surgical cavity of recurrent GBM patients is well established and provides discernable survival benefits^1,3,10,11^, while bearing several known potential hazards, mainly consisting of wound healing problems and infections^13,14^. This study, for the first time, assesses the dynamics of several tumor characteristics after Gliadel implantation over time. It is clearly apparent and statistically significant that increasing volumes of FA, TB, and RC negatively affect survival. The expansion of TB’s volume carrying a dismal prognosis, sometimes with no further clinical manifestations, could imply disease progression according to RANO criteria^26^. Increasing volume of FA could also reflect oncological worsening^26,27^, yet the prognostic significance of an abnormality when FLAIR had been measured volumetrically over time lost its significance in a multivariate analysis.

The volume of the RC also emerged as being a significant negative predictor of survival in both univariate and all-inclusive multivariate analyses. We postulate that this finding is related in some patients to the formation of post-surgical cysts and in other cases to tumor activity causing necrosis that is eventually seen as an expanding surgical cavity. Tumor activity is directly observed by measuring the TB and, indeed, a stepwise multivariate analysis demonstrated that TB was the only parameter significantly predicting survival, explaining 73% of the variance in survival between patients.

In an attempt to account for the biological activity that Gliadel might induce in the tissue, we investigated the subgroup of patients with a reactive FLAIR image at 3–8 weeks which subsequently decreased. This timeframe was determined based upon the understanding that Gliadel's immunological influence over the brain parenchyma will have occurred and ceased during this timeframe^17^. The threshold for increase and decrease in FLAIR volume was set at 20% to confirm that a true change had been recorded and that the values were not merely errors in measurement. This threshold complies with the reported difference of volumetric assessments as being at around 10–15%^23,26,28^. It should be noted that there are currently no standards for assessing FLAIR abnormalities (in contrast to gadolinium enhanced T1 weighted MRI measurements which are assessed using the RANO criteria). The possible resolution of postoperative edema (and associated FA) with the use of steroids is accounted for by the routine use of steroids (regularly slowly tapered down) among all patients treated with Gliadel.

Comparing this subgroup of patients to the rest of the cohort, we showed a significant difference in survival between these patient groups, with triple the survival period after the Gliadel implantation surgery for patients with FLAIR reactivity. This finding did not appear as being significant in the multivariate analysis (*p* = 0.28), probably due to the group’s small size (of six patients) and consequent lack of power.

Limitations of this study include the fact it was done in a retrospective fashion and on a rather small (n = 56) cohort (of which derived even smaller subgroup of FLAIR reactivity, n = 6). In addition, some clinical and radiological data were missing (especially the immediately postoperative imaging studies). Additionally, molecular data and MGMT methylation status were also not available for the entire cohort. Adjuvant therapies were given to 39 patients (70%), which might confound our findings.

## Conclusions

Increasing volumes of FA, TB, and RC negatively affect overall survival. TB was more prominent parameter than other measured clinical and radiological parameters, Transient peritumoral edema, as evident by FLAIR abnormality following Gliadel implantation, might reflect anti-tumor activity and was found to be significant predictor for longer GS, reaching triple that of the rest of the cohort. Validating these findings for clinical application requires further research, perhaps with shorter intervals between imaging tests that will allow the assessments of tumoral activities and changes in treatment planning based on them.

## Data Availability

The raw data including dataset and volumetric measurements is secured and saved in Tel-Aviv Medical Center servers and will be accessible upon requirements, according to evaluation of necessity by IRB. Appliances should be sent by email to the corresponding author—Rachel Grossman (rachelyg@hotmail.com) and to the authors—Oz Haim and Ariel Agur (ozi.haim@gmail.com and ariel@agur.im respectively).
